# CCL20/MIP-3 alpha mRNA expression in the conjunctival epithelium of normal individuals and patients with vernal keratoconjunctivitis

**DOI:** 10.1007/s00417-014-2785-1

**Published:** 2014-08-30

**Authors:** Noriko Inada, Akiko Ishimori, Jun Shoji

**Affiliations:** Division of Ophthalmology, Department of Visual Sciences, Nihon University School of Medicine, 30-1 Oyaguchi-Kamichou, Itabashi-ku, Tokyo 173-8610 Japan

**Keywords:** Allergy, CCL20, Conjunctival epithelium, Impression cytology, Vernal keratoconjunctivitis

## Abstract

**Background:**

CCL20, the single chemokine ligand for CCR6, contributes to recruiting CCR6-expressing memory B cells, memory T cells, Th17 cells and dendritic cells, and is involved in regulating immune responses, homeostasis, and inflammation in mucosal tissues.

**Methods:**

CCL20 messenger RNA (mRNA) expression was analyzed in the conjunctival epithelium in an in vivo study of patients with vernal keratoconjunctivitis (VKC group) and healthy volunteers (control group) using impression cytology. In vitro analysis of CCL20 mRNA was performed using cultured conjunctival epithelial cells (CECs). Real-time polymerase chain reaction was used to assess IL-8 and eotaxin-2 mRNA expression for comparison with CCL20 mRNA expression.

**Results:**

In the control group, CCL20 mRNA expression was present in all conjunctival locations. However, CCL20 mRNA expression was significantly higher in the upper palpebral conjunctiva in the severe VKC group than in the mild VKC and control groups (*p* < 0.05, Steel test). In vitro stimulation of CECs with lipopolysaccharide (LPS) significantly increased CCL20 expression in a concentration-dependent manner that was significantly correlated with expression of IL-8 (*p* < 0.001, Spearman’s rank correlation coefficient), but not eotaxin-2.

**Conclusion:**

We conclude that CCL 20 mRNA expression in the conjunctival epithelium plays a crucial role in regulating homeostasis at the ocular surface and in exacerbation of VKC.

## Introduction

Chemokines are involved in acute and chronic inflammatory processes, as they attract neutrophils, monocytes, B cells, and T cells to the inflammation site via their corresponding chemokine receptors. Recent reports have shown that the conjunctival epithelium is an important chemokine producer under pathophysiologic conditions observed in ocular surface diseases [1, 2]. In allergic conjunctival diseases, eotaxin-1 and eotaxin-2 expression in the conjunctival epithelium is crucial for inducing allergic inflammation in the conjunctiva through eosinophil recruitment [3]. Furthermore, conjunctival epithelial cells contribute to the pathogenesis of ocular surface diseases by producing proinflammatory cytokines such as interleukin (IL)-8 [4, 5]. IL-6 and IL-8 can be detected in the tears of healthy individuals [6]. Thus, chemokines function as inflammatory and homeostatic factors at the ocular surface. CCL20/macrophage inflammatory protein-3 alpha (MIP-3α) has recently been identified as an important chemokine in the regulation of homeostasis and inflammation in mucosal tissues. CCL20, which is the single chemokine ligand for CCR6, contributes to these processes by recruiting CCR6-expressing memory B cells [7–9], CD45RO^+^ memory T cells [10], IL-17-secreting CD4^+^ T cells (Th17) [11], and dendritic cells such as Langerhans cells [12] .

Proliferative conjunctival lesions such as giant papillae are an important feature of severe allergic conjunctival diseases. Histological analysis of proliferative conjunctival lesions has demonstrated infiltration of many inflammatory cells such as neutrophils, eosinophils, mast cells, and CD45RO^+^ memory T cells [13] in subconjunctival tissues in such diseases. Multiple stimuli induce chemokine expression in the conjunctival epithelium. Chemokines in the conjunctiva, including eotaxin-1, eotaxin-2, and eotaxin-3 induce eosinophilic infiltration, whereas IL-8 induces neutrophilic infiltration. CCL20 has been reported to be involved in Th17 cell invasion of the ocular surface in a dry eye mouse model [14]. Data from this mouse model suggest that CCL20 is associated with conjunctival inflammation. However, the mechanisms underlying CCL20 expression in the human conjunctival epithelium in patients with allergic conjunctival diseases remain unknown.

This study aimed to investigate CCL20 mRNA expression in the human conjunctival epithelium in vivo by using impression cytology specimens from healthy individuals and patients with vernal keratoconjunctivitis (VKC). In addition, an in vitro study analyzing CCL20 mRNA expression in cultured conjunctival epithelial cells (CECs) was performed.

### Subjects and methods

This study was approved by the Nihon University School of Medicine’s Ethics Committee for Clinical Study and adhered to the tenets of the Declaration of Helsinki. The experimental design of the clinical study consisted of an in vivo study using impression cytology specimens and an in vitro study using cultured CECs.

### Analysis of in vivo CCL20/MIP-3α mRNA expression in the conjunctival epithelium of healthy volunteers and patients with vernal keratoconjunctivitis

#### Subjects

The subjects comprised 11 healthy volunteers (eight men and eight women; mean ± SD of age, 30.6 ± 8.6 years) (control group) and 11 patients who had VKC (11 men; mean ± SD of age, 23.1 ± 10.4 years) (VKC group) and had been treated at the Department of Ophthalmology, Nihon University Itabashi Hospital, from June 2009 to July 2010. Demographic and clinical data of patients with VKC are shown in Table 1. According to the Japanese guidelines [15], we diagnosed VKC in patients with both of proliferative conjunctival lesions such as giant papillae or gelatinous limbal infiltration and allergen-specific IgE antibodies in serum at initial diagnosis. The enrolled VKC patients had one or several positive allergen-specific IgE antibodies, such as those to house dust mites or Japanese cedar pollen, detected in their serum using ImmunoCAP® specific IgE (Thermo Scientific, Yokohama, Japan). Patients who had VKC and had a history of systemic corticosteroid and/or immunosuppressive drug treatment were excluded from the study. Informed consent for participation was obtained before enrolment. Conjunctival impression cytology for the VKC group was performed for either the affected eye in unilateral cases or the more severely affected eye in bilateral cases. Healthy volunteers (11 right eyes) who had neither allergic diathesis nor a history of wearing contact lenses were also recruited as controls.

**Table 1:** Demographic and clinical data of patients with VKC

Case	Age (year)	Sex	Type of VKC	Clinical score*	Severity	Stage of VKC	Complication of AD	Therapeutic medicine
1	30	Male	Mix	223	severe	Active	+	DSCG, TA
2	20	Male	Palpebral	22	Mild	Stable	+	DSCG, TA
3	7	Male	Mix	113	Moderate	Active	+	DSCG, TA
4	34	Male	Mix	23	Mild	Stable	+	DSCG, TA
5	41	Male	Palpebral	113	Moderate	Stable		DSCG, CsA
6	21	Male	Palpebral	23	Mild	Stable		DSCG, TA
7	27	Male	Mix	23	Mild	Stable	+	DSCG, TA
8	30	Male	Palpebral	223	severe	Active	+	DSCG, TA
9	19	Male	Mix	124	Moderate	Active	+	DSCG, TA
10	9	Male	Palpebral	112	Moderate	Stable		None
11	16	Male	Mix	123	Moderate	Active		DSCG, TA

*: 5-5-5 exacerbation grading scale [reference 16)]

AD: atopic dermatitis, VKC: vernal keratoconjunctivitis, DSCG: disodium cromoglycate ophthalmic solution, TA: Tacrolimus ophthalmic solution, CsA: Cyclosporine A ophthalmic solution

#### CCL20 mRNA expression in the conjunctival epithelium of healthy subjects and patients with VKC

In the first study, we investigated the differences in the conjunctival localization of CCL20 mRNA expression in the different control subjects. Impression cytology was performed for the upper palpebral conjunctiva, lower palpebral conjunctiva, and temporal bulbar conjunctiva, and local differences in the CCL20 mRNA level were compared among all control subjects.

In the second study, we investigated the differences in CCL20 mRNA expression induced by allergic inflammation between for the VKC and control groups. In the VKC group, impression cytology was performed for the upper palpebral conjunctiva, and pathological differences in the CCL20 mRNA level were compared between the VKC and control groups.

#### Scoring of clinical severity in VKC patients

The clinical severity in VKC group patients was scored using the 5-5-5 exacerbation grading scale [16]. The 5-5-5 exacerbation grading scale has been previously reported as a method for measuring the severity of allergic conjunctival diseases. With this scoring method, the clinical score is determined by the total number of points. Five severe clinical findings are given 100 points for each finding, five moderate clinical findings are given 10 points for each finding, and five mild clinical findings are given 1 point for each finding (Table 2). Using this method, the patients with VKC were divided into severe, moderate, and mild subgroups. Based on the 5-5-5 exacerbation grading scale, the VKC patients were divided into three phases of clinical severity: severe (more than 200 points), moderate (100–200 points) and mild (less than 100 points). Because of the low overall number of cases, the evaluated VKC subgroup was divided into the following two subgroups: the severe subgroup consisted of VKC patients with both severe and moderate clinical severity, and the mild subgroup consisted of VKC patients with mild clinical severity.

**Table 2:** Guidelines for the 5-5-5 exacerbation grading scale used to score allergic conjunctival diseases

Exacerbation grading scale *			
Grade of Clinical sign	100-point grade	10-point grade	1-point grade
	Active giant papillae ^1)^	Blepharitis	Papillae at the upper palpebral conjunctiva
	Gelatinous infiltrates of the limbus	Papillary proliferation with velvety appearance	Follicular lesion at the lower palpebral conjunctiva
Clinical signs	Exfoliative epithelial keratopathy	Horner-Trantas spots	Hyperemia of the palpebral conjunctiva
	Shield ulcer	Edema of the bulbal conjunctiva	Hyperemia of the bulbal conjunctiva
	Papillary proliferation at the lower palpebral conjunctiva	Superficial punctate keratopathy	Lacrimal effusion ^2)^
Score	100 points × number of positive signs	10 points × number of positive signs	1 point × number of positive signs
Range	0–500 points	0–50 points	0–5 points

*: 5-5-5 exacerbation grading scale [16]

1) “Active giant papillae” means the giant papillary proliferation that spreads in hemispherical shape with mucous discharge, but mucous discharge is not essential. Giant papillae with flatness and poor inflammation findings are excluded

2) “Lacrimal effusion” refers to the epiphora or tear meniscus increase caused by eye irritation

#### Impression cytology

Impression cytology was performed after an instillation of topical 0.4 % oxybuprocaine (Benoxil; Santen, Osaka, Japan). Strips of nitrocellulose membranes (Millipore, Bedford, MA, USA) were applied to the conjunctiva, pressed gently by a glass rod, and then removed. The nitrocellulose membrane was then preserved in RNAlater RNA Stabilization Reagent (Qiagen, Hilden, Germany) until analysis.

#### Real-time polymerase chain reaction

For detection of CCL20 mRNA expression by real-time polymerase chain reaction (real-time PCR), total RNA from each impression cytology specimen was harvested using an RNeasy® Mini Kit (QIAGEN, Hilden, Germany) by following the instructions in the manufacturer’s manual. cDNA was then synthesized using a High-Capacity cDNA Reverse Transcription Kit (Life Technologies Japan, Tokyo, Japan) according to the manufacturer’s instructions.

Real-time PCR was performed using a commercial PCR master mix (TaqMan Universal PCR Master Mix; Life Technologies Japan) and predesigned primers (Life Technologies Japan) for CCL20 (Hs00355476_m1), IL-8 (Hs99999034_m1), and eotaxin-2 (Hs00171082_m1). Samples were analyzed using a real-time PCR system (Step One Plus^TM^; Life Technologies Japan) and comparative threshold (Ct) values were obtained. Target Ct values were normalized to those of GAPDH (Hs99999905_m1) from the same sample. Data were analyzed by the ∆∆CT method.

#### Statistical analysis

CCL20 mRNA expression in impression cytology specimens was evaluated by the nonparametric Steel-Dwass test for investigation of differences in conjunctival localization changes (first study), and the Steel test was used to investigate of differences in allergic inflammation (second study). We evaluated the correlation between CCL20 mRNA and IL-8 mRNA levels and that between CCL20 mRNA and eotaxin-2 mRNA levels by using a nonparametric correlation method, the Spearman correlation coefficients. *p* <0.05 was considered to indicate statistical significance.

#### In vitro study evaluating CCL20/MIP-3α mRNA expression in cultured human conjunctival epithelial cells

##### Human conjunctival epithelial cell culture

A human conjunctival epithelial cell line (Wong-Kilbourne derivative of Chang conjunctiva, clone 1-5c-4, CCL-20.2; American Type Culture Collection [ATCC], Manassas, VA, USA) was cultured under standard conditions (humidified atmosphere of 5 % CO2 at 37 °C) in a mixed medium containing Ham’s F12 and Dulbecco’s modified Eagle’s medium (DMEM; 1:1; Life Technologies Japan) supplemented with recombinant epidermal growth factor (10 ng/mL; Life Technologies Japan), recombinant insulin (5 μg/mL; Life Technologies Japan), dimethyl sulfoxide (0.5 %; Sigma-Aldrich Japan, Tokyo, Japan), gentamicin (40 μg/mL; Schering-Plough, Osaka, Japan), penicillin G (100 U/mL; MSD, Tokyo, Japan), and 10 % fetal bovine serum (Life Technologies Japan) in 35-mm tissue culture dishes (Falcon 3001; Becton Dickinson, Tokyo, Japan). The cultured CECs were detached after incubation in 0.25 % trypsin and 0.5 % EDTA (Sigma-Aldrich Japan) after they reached confluency. The resuspended cells were then seeded in 24-well plates (ASAHI GLASS, Tokyo, Japan) at 10^4^ cells per well and cultured until they reached confluence. Confluent CECs were then exposed to various concentrations of lipopolysaccharide (LPS) (0, 20, 40, 80, and 160 μg/mL) for 4 h and analyzed for mRNA expression.

### Real-time PCR

Real-time PCR for CCL20 mRNA expression in human cultured CECs was performed using the same method described above.

### Statistical analysis

CCL20 mRNA expression in cultured human CECs were evaluated using the nonparametric Steel test. Spearman correlation coefficients were used to evaluate whether CCL20 mRNA expression was correlated with IL-8 mRNA expression. *p* <0.05 was regarded as statistically significant.

## Results

### CCL20 mRNA expression in the conjunctival epithelium in the control group

**Fig. 1:**
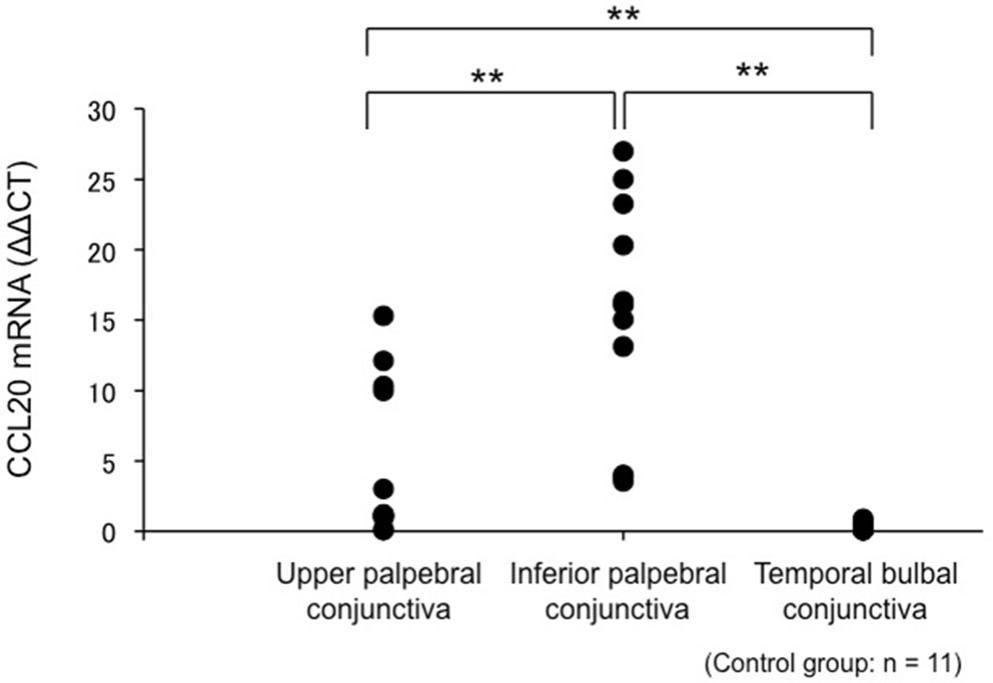
CCL20 mRNA expression in the upper palpebral, lower palpebral, and temporal bulbal conjunctiva. CCL20 mRNA expression was high in the lower palpebral conjunctiva, upper palpebral conjunctiva, and temporal bulbar conjunctiva. In particular, CCL20 mRNA expression was significantly higher in the lower palpebral conjunctiva than in the upper palpebral conjunctiva and temporal bulbar conjunctiva. **: Steel-Dwass test, *p* < 0.01

CCL20 mRNA expression in healthy volunteers was detected in impression cytology specimens obtained from the upper palpebral, lower palpebral, and temporal bulbal conjunctiva. The (median [range]) CCL20 mRNA level in the upper palpebral conjunctiva, lower palpebral conjunctiva, and temporal bulbal conjunctiva was 1.20 (0.10–15.3), 16.1 (3.54–27.0), and 0.18 (0.08–0.89), respectively. CCL20 mRNA expression in the lower palpebral conjunctiva was significantly higher than that in the upper palpebral conjunctiva (Steel-Dwass test, *p* < 0.01; Fig. 1). Furthermore, the CCL20 mRNA levels in the upper palpebral conjunctiva were significantly higher than those in the temporal bulbal conjunctiva (Steel-Dwass test, *p* < 0.01; Fig. 1).

### CCL20 mRNA expression in the upper tarsal conjunctiva in the VKC and control groups

**Fig. 2:**
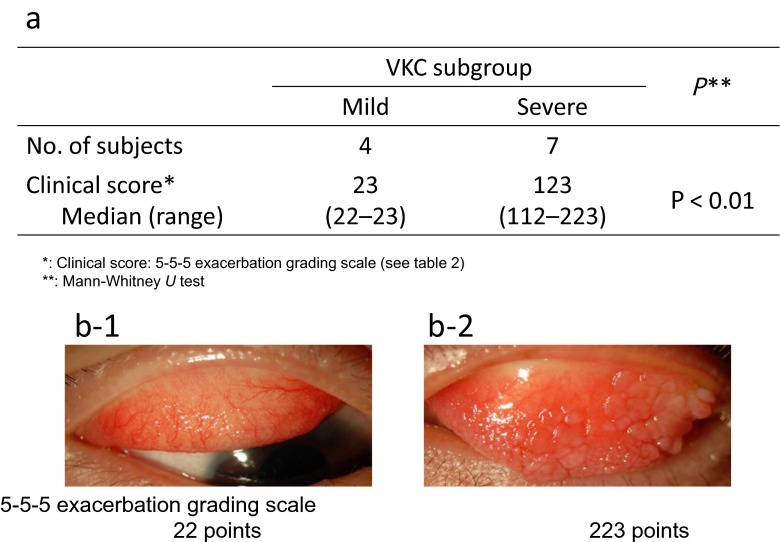
Vernal keratoconjunctivitis (VKC) subgroup. a: Characteristics of the VKC subgroups. b: Representative photograph of a patient with mild VKC (b-1) and a patient with severe VKC (b-2). The criterion for severe VKC was a clinical score greater than 100 points and for mild VKC was a clinical score lower than 100 points

**Fig. 3:**
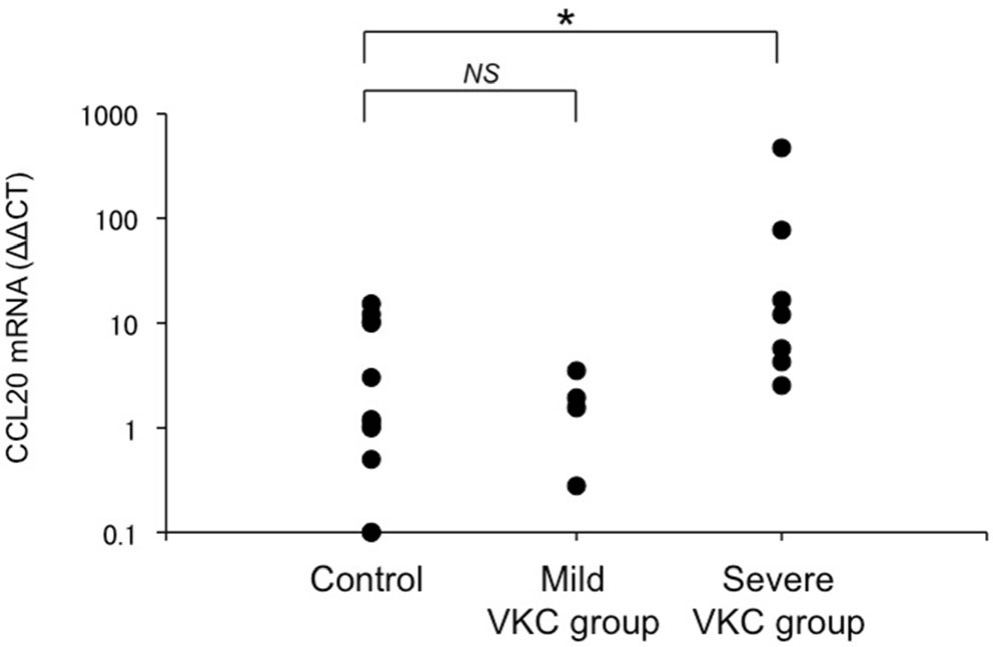
CCL20 mRNA expression in the control and VKC groups. In the upper palpebral conjunctiva, CCL20 mRNA expression was significantly higher in the severe VKC group than in the control group (*p* < 0.05, Steel test). CCL20 mRNA expression did not significantly differ between the control group and mild VKC group. *: *p* < 0.05, Steel test. NS: not significant

The VKC group was divided into the severe VKC and mild VKC subgroups according to the clinical score (Table 1, Fig. 2). The (median [range]) CCL20 mRNA levels in the upper palpebral conjunctiva in the mild and severe VKC subgroups were 2.52 (0.28–5.70) and 46.7 (12.0–471), respectively. CCL20 mRNA expression in the severe VKC group was significantly higher than that in the control group (Steel test, *p* < 0.05; Fig. 3).

**Fig. 4:**
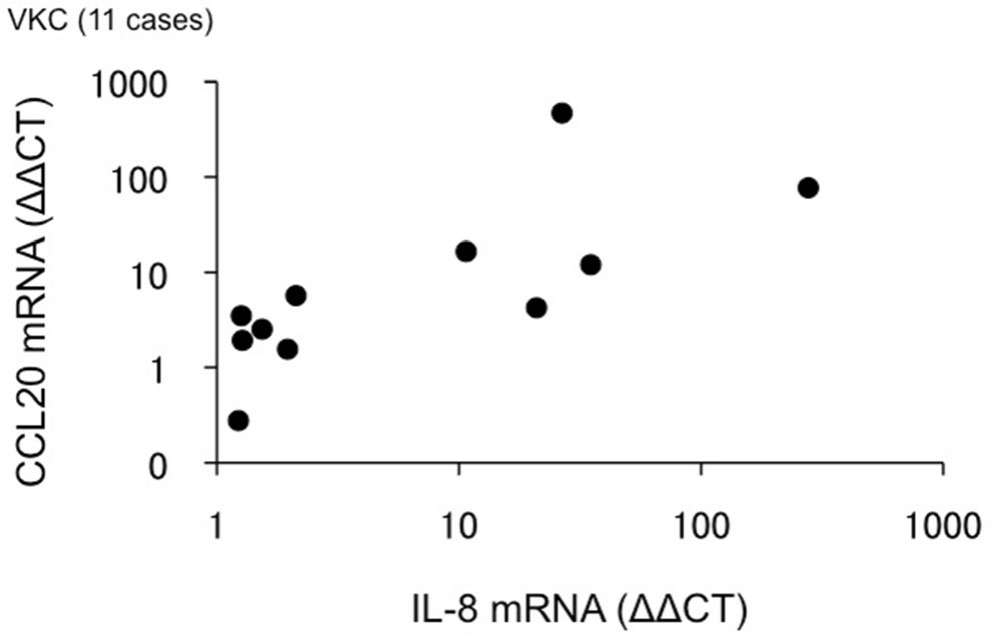
Correlation between CCL20 mRNA expression and IL-8 mRNA expression in the VKC group. CCL20 mRNA expression was significantly correlated with IL-8 mRNA expression in the conjunctiva (*r* = 0.83, *p* < 0.01, Spearman’s rank correlation coefficient)

The CCL20, IL-8, and eotaxin-2 mRNA levels obtained in the VKC group are shown in Table 3. The CCL20 mRNA levels were significantly correlated with IL-8 mRNA levels (Spearman’s correlation, *r* = 0.83, *p* < 0.01; Fig. 4), but not with eotaxin-2 mRNA levels.

**Table 3:** CCL20, IL-8, and eotaxin-2 mRNA expression in the VKC group

	mRNA expression (ΔΔCT) Median (range)
CCL20	4.24 (0.28–471)
IL-8	2.12 (1.23–277)
Eotaxin-2	3.01 (0.10–26.8)

### CCL20 mRNA expression in cultured conjunctival epithelial cells

**Fig. 5:**
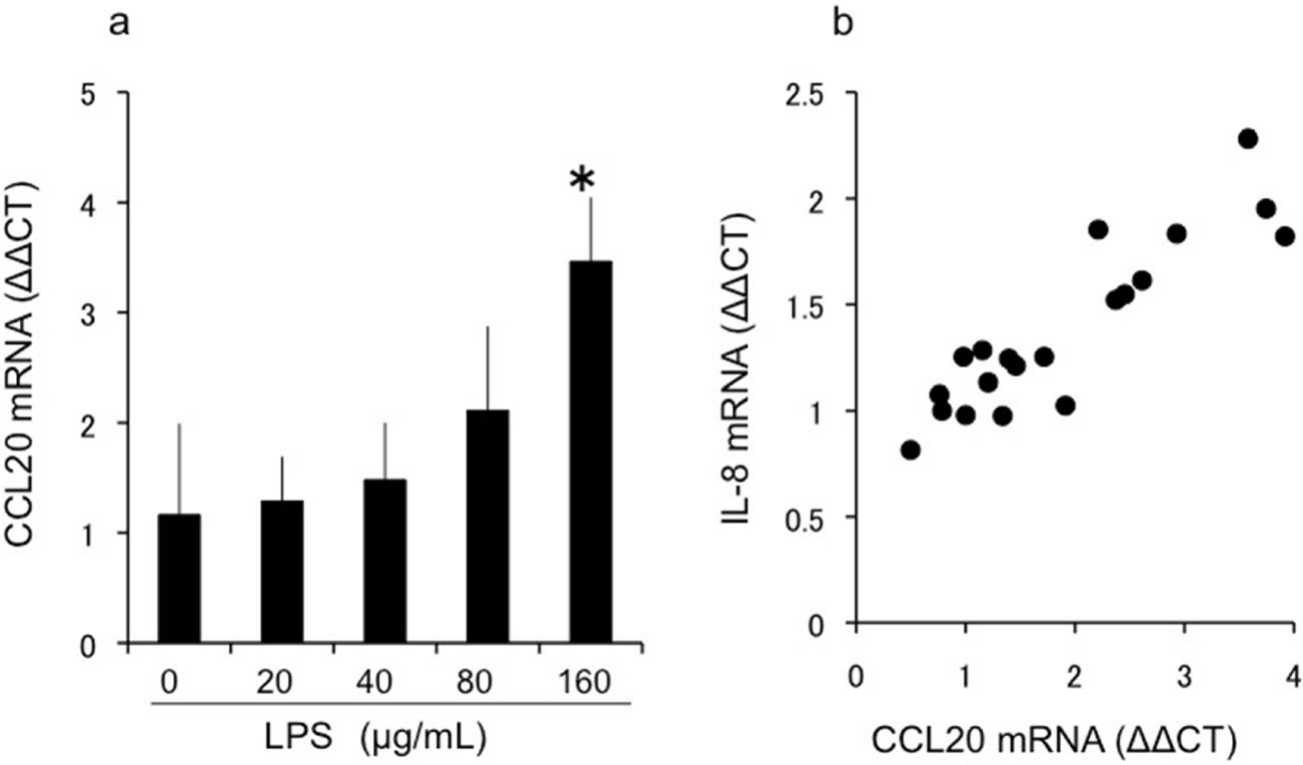
CCL20 mRNA expression in cultured conjunctival epithelial cells. a: In the cultured conjunctival epithelial cells, CCL20 mRNA expression increased in a dose-dependent manner in response to lipopolysaccharide (LPS) stimulation (*p* < 0.05, Steel test). b: In the cultured conjunctival epithelial cells stimulated with LPS, CCL20 mRNA expression was significantly correlated with IL-8 mRNA expression (*r* = 0.82, *p* < 0.001, Spearman’s rank correlation coefficient)

Analysis of CCL20 mRNA expression in LPS-stimulated CECs indicated a significant concentration-dependent increase in CCL20 expression induced by LPS (Fig. 5a), which was correlated with IL-8 mRNA expression (Fig. 5b).

## Discussion

CCL20 is a C-C chemokine associated with the migration of CCR6-expressing inflammatory cells such as dendritic cells, B cells, memory T cells, and Th17 cells. These CCR6-positive immunocytes are thought to be involved in immune responses, homeostasis [17], and inflammation [18] in mucosal tissues [19]. Therefore, evaluation of local CCL20 expression in the ocular surface is thought to be important for understanding the pathologic features of inflammatory conjunctival diseases, including infectious conjunctivitis and allergic conjunctival diseases.

Initially, we assessed CCL20 mRNA expression in the ocular surface of healthy volunteers by using the impression cytology method. Because biopsy samples including superficial epithelial cells of the ocular surface can be obtained with this method, this is the most useful method for analyzing the pathologic conditions of the local conjunctiva, as it enables the measurement of chemokine mRNA expression in cytology specimens. Our results showed that CCL20 mRNA is constitutively present in the conjunctival epithelium. These results agree with those of previous reports demonstrating that CCL20 is involved in mucosal homeostatic maintenance [17].

We also found that CCL20 mRNA expression levels were higher in the palpebral conjunctiva than in the bulbar conjunctiva. In previous studies, CCL20 expression in the intestinal epithelium has been reported to strongly increase in the lymphoepithelium and M cells of the Peyer’s patch [20]. Alternatively, human conjunctiva-associated lymphoid tissue (CALT), which is functionally equivalent to Peyer’s patches, is present mainly in the palpebral conjunctiva [21, 22]. M-cell-like cells have also been shown to be present in the epithelium covering CALT [23, 24]. Furthermore, the conjunctival follicles formed mainly on lower palpebral conjunctiva have characteristics similar to those of CALT. Therefore, in our study, the anatomical characteristics of the conjunctiva may have influenced CCL20 mRNA expression in the palpebral conjunctiva, resulting in higher levels of CCL20 expression than those in the bulbar conjunctiva.

In our study, we assessed CCL20 mRNA expression in the upper palpebral conjunctiva where papillary proliferation is observed in patients with VKC. CCL20 mRNA expression in the upper palpebral conjunctiva was significantly higher in the severe VKC group than in the control group. These results show that CCL20 is constantly expressed in the conjunctival epithelium and that its expression increases when allergic inflammation in the conjunctiva is aggravated. Several reports have suggested that inflammatory cells express CCR6, which is a ligand of CCL20 associated with allergic inflammation [25]. Lukacs et al. reported that CCR6-deficient mice had reduced airway resistance, fewer eosinophils around the airway, lower interleukin-5 levels in the lung, and reduced serum IgE levels [26]. Furthermore, local expression of CCL20 in allergic and immunological disorders has been demonstrated by Reibman et al., who showed that airway epithelial cells release CCL20/MIP-3 alpha in response to cytokines and ambient particulate matter in cultured human bronchial airway epithelial cells [27]. Furthermore, increased CCL20 levels have been shown in sputum obtained from patients with severe asthma. Increased CCL20 expression in keratinocytes of patients with psoriasis has been reported, and the contribution of CCR6-positive Th17 cells to the pathology of inflammatory diseases has been suggested clinically [28, 29]. Therefore, the CCL20/CCR6 axis is thought to be an important local factor involved in regulating the pathology of allergic inflammation in patients with severe VKC.

The conjunctival epithelium produces various chemokines as a defence mechanism against antigen invasion from the external environment. IL-8, a proinflammatory cytokine produced by the conjunctival epithelium [4], is involved in biophylaxis and induces neutrophilic migration. In addition, eotaxin is known to be an important chemokine in the pathology of allergic reactions at the ocular surface. Eotaxin-1/CCL11, eotaxin-2/CCL24, and eotaxin-3/CCL26 are part of the eotaxin subfamily, and each eotaxin subfamily induces eosinophilic migration through CCR3. We had previously reported that eotaxin-2 levels are significantly increased in the tears of patients with VKC and that this increase is significantly correlated with the concentration of eosinophil cationic protein in tears [3]. Our in vivo study on the conjunctival epithelium of patients with VKC indicated that CCL20 expression was significantly correlated with the expression of IL-8, but not eotaxin-2. The results of this in vivo study were validated by the in vitro study using cultured conjunctival epithelial cells. To stimulate the conjunctival epithelial cells, we used LPS, which induces IL-8 production and is an endotoxin found in the outer membrane of gram-negative bacteria. CCL20 mRNA levels were significantly correlated with IL-8 levels, similar to the in vivo results. These results demonstrate that normal flora in the conjunctival sac and pathogenic bacteria of infectious keratoconjunctivitis may be involved in regulating CCL20 expression in the conjunctival epithelium. Epithelial CCL20 expression in the intestinal epithelia is reported to increase in response to LPS stimulation [30]. Therefore, the CCL20/CCR6 axis may be strongly associated with exacerbation of VKC in response to components of the external environment, including bacteria.

Further investigation is necessary to enable practical use of CCL20 mRNA expression in superior palpebral conjunctiva as a biomarker of inflammatory or infectious conjunctival diseases.

Therefore, we conclude that CCL20 mRNA expression in the conjunctival epithelium plays a crucial role in homeostasis at the ocular surface and in exacerbation of VKC. Further studies are necessary to determine the molecular mechanism by which CCL20 expression in the conjunctival epithelium is enhanced by the external environment.
